# Iron deficiency in a tertiary gastroenterology center in Romania: prevalence and significancy

**Published:** 2018

**Authors:** Carmen Monica Preda, Doina Proca, Irina Sandra, Boroka Claudia Horeanga, Larisa Elena Fulger, Teodora Manuc, Ion Bancila, Oana Elena Balas, Mircea Manuc, Mircea Diculescu, Cristian Baicus, Cristian Tieranu, Ileana Constantinescu

**Affiliations:** * Gastroenterology&Hepatology Department, Clinic Fundeni Institute, Bucharest, Romania; ** Internal Medicine Department, Colentina Hospital, Bucharest, Romania; *** Gastroenterology&Hepatology Department, Elias Hospital, Bucharest, Romania; **** Centre for Immunogenetics and Virology, Clinic Fundeni Institute, Bucharest, Romania

**Keywords:** Absolute iron deficiency, Relative iron deficiency, Iron-deficiency anemia

## Abstract

**Introduction:**Iron deficiency has been known to cause significant functional impairment, lower quality of life and higher morbidity and mortality. The aim of this study was to estimate the prevalence and significance of iron deficiency in our patients and medical staff.

**Material and methods:**We performed a prospective cross-sectional study: In July 2016, 383 persons were screened for the presence of iron deficiency (ID): 325 patients and 58 people from the medical staff. Transferrin saturation (TSAT), serum ferritin (SF) and complete blood count were performed. Absolute ID was diagnosed if SF <100 ng/ml and TSAT <20%. Relative ID was defined by SF >100 ng/ml and TSAT <20%.

**Results:**The group of medical staff was younger and had a greater proportion of women. The prevalence of absolute ID was 22.5% in patients and 43.1% in medical staff; relative ID was present in 15% of patients and 1.7% of medical staff. Among patients, the absolute ID was significantly correlated with the female sex (p=0.002) and pre-menopausal status (p=0.01) but did not correlate with diagnosis, age, BMI, nonsteroidal anti-inflammatory drug (NSAID), aspirin or acenocoumarol consumption. The relative ID is associated with advanced age (p=0.03) and diagnosis of cancer and liver cirrhosis (p=0.01).

**Conclusions:**Absolute ID had a high prevalence among patients (22.5%), but there was even a bigger issue among the medical staff (43.1%). Absolute ID was correlated with female sex and pre-menopausal status. Relative ID was related to advanced age, cancer and liver cirrhosis.

**Abbreviations:** serum ferritine- SF, transferrin saturation coefficient- TSAT, iron deficiency- ID, inflammatory bowel diseases- IBD, quality of life- QoL, GI- gastrointestinal

## Introduction

Anemia is a global health concern of epidemic proportions and the most common blood disorder affecting 1 in 3 adults worldwide according to a study published in 2016. Iron deficiency is the leading cause, responsible for more than 60% of anemias [**1**]. A 2013 World Health Organisation report stated that anemia and iron deficiency cause physical impairment and reduction in working capacity in adults, influencing social progress [**2**].

Hospitalised patients are at greater risk of developing iron deficiency anemia with a poorer outcome, depending on their base condition and comorbidities [**3**].

Iron deficiency anemia comprises 3 stages: depleted iron stores (with normal erythropoiesis in contrast to the other stages), iron deficient erythropoiesis (still with normal levels of hemoglobin) and iron deficiency anemia [**4**].

Iron-restricted erythropoiesis occurs in cases of both absolute and functional iron deficiency. The latter setting represents a state of iron-restricted erythropoiesis characterized by an imbalance between iron demand and serum iron that is readily available for effective erythropoiesis [**5**].

Diagnosis of iron-restricted erythropoiesis is based on the use of widely available standard biochemical parameters: serum iron, transferrin saturation (TSAT), and ferritin [**5**].

The normal value of serum ferritin is 100÷150 ng/ml in women and 100÷400 ng/ml in men. The normal reference range for TSAT is 20-45%. The serum iron reference range is 55–160 µg/dL in men and 40–155 µg/dL in women.

The cause of functional iron deficiency is the inflammation. Inflammation impairs the utilization of ferritin necessary for normal erythropoiesis. Therefore, administration of intravenous iron, in this case, might overcome this barrier and lead to effective erythropoiesis. The molecular mechanisms that affect hepcidin and erythroferrone regulation in the redistribution of iron stores during inflammatory processes are not fully understood, although their dysregulation seems to be responsible for ineffective erythropoiesis [**6**].

Gastrointestinal diseases are the main cause of iron deficiency in men and elderly, involving three mechanisms: malnutrition, malabsorption and increased losses of iron through bleeding [**7**][**8**]. The prevalence of iron deficiency related to malabsorption varies within a broad range, from 14% to 81%, according to the study and comorbidities (inflammatory bowel disease, celiac disease or chronic gastritis) [**9**][**10**][**11**] whereas bleeding causes anemia in 2.5-47.5% of the cases (in Crohn’s disease, colorectal cancers, ulcer) [**12**][**13**][**14**].

The prevalence of iron deficiency in patients with heart failure is around 50% [**15**], and it can reach 73% in patients with NYHA class IV heart failure [**16**]. Iron deficiency has been known to cause significant mitochondrial malfunction [**17**] and tissue remodeling (cardiac fibrosis), resulting in functional impairment, lower quality of life and higher morbidity and mortality. Iron deficiency, with or without anemia has proved to be a strong and independent predictor of mortality [**15**].

In patients that undergo major surgery, preoperative anemia has a prevalence of 75%, and it may be added to the surgical risks [**18**][**19**].

Screening for iron deficiency by determining serum ferritin and transferrin saturation coefficient is recommended by the European Society of Cardiology 2012 guidelines in patients with heart failure [**20**] and European Crohn’s and Colitis Organization 2015 guidelines in patients with inflammatory bowel diseases [**21**]. The objective of the study was to determine the prevalence of iron deficiency in adult patients hospitalized in the Department of Gastroenterology II, Fundeni Clinical Institute and our medical staff over a two-week-long period and factors associated with iron deficiency.

## Materials and methods

We performed a cross-sectional study, consisting in the collection of blood samples after signing an informed consent from all the patients hospitalized in the Department of Gastroenterology II, Fundeni Clinical Institute, Bucharest, Romania and all the medical staff from the same Department, during 2 weeks in July 2016. The biochemical parameters recommended by several guidelines such as serum ferritin, transferrin saturation coefficient, serum iron and complete blood count were determined. Blood samples were analyzed in an external laboratory (Synevo) with the financial support of Vifor Pharma Romania. Vifor Pharma Romania had no other involvement or control over the conduct of this research, excepting the above-mentioned support in determining the biochemical parameters.

In order to characterize the population included in the study, we have collected information regarding demographics and clinical data : BMI, diagnosis, NSAID, aspirin and acenocoumarol consumption, pre-or postmenopausal status for women.

**Inclusion criteria:**

All adult patients (age above 18 years old) hospitalized for 2 weeks in July 2016 were included in the research, regardless of comorbidity: 325, with one-day hospitalization or continuous hospitalization. 58 persons from the medical staff also had their blood tested.

**Fig. 1:**
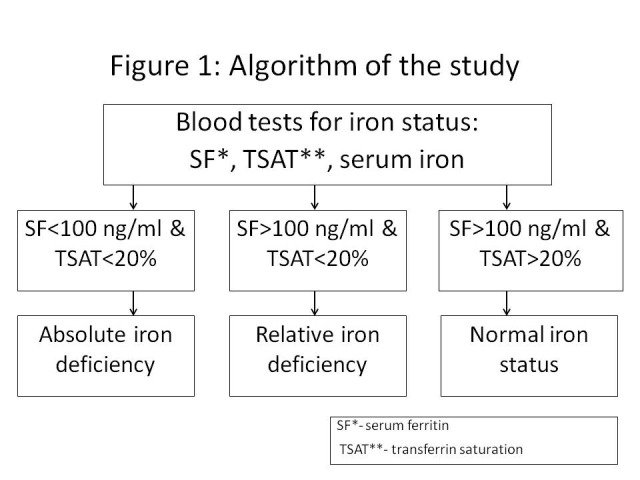
Algorithm of the study

The absolute iron deficiency was diagnosed if serum ferritin (SF) <100 ng/ml and TSAT <20% [28, 31] (**Figure 1**).

The relative iron deficiency was defined by SF >100 ng/ml and TSAT <20% [28, 31] (**Figure 1**).

Anemia was defined as hemoglobin level of less than 13.5 g/dl in men and less than 12 g/dl in women [43].

**Exclusion criteria:**

Children, critically ill patients, and those who did not wish to provide or could not provide informed consent.

The study was approved by the Ethics Committee of Fundeni Clinical Institute.

**Statistical analysis:**

Data analysis was performed using the statistical software SPSS 16.0 ( SPSS, Inc., Chicago, IL, USA). Categorical variables were reported as frequency and analyzed using Fisher’s exact test. Continuous variables that were not normally distributed were reported as median (minimum to maximum) and analyzed using the Mann-Whitney U test.

## Results

The whole group is 67% females, with a median age of 55 (17÷88), a median BMI of 25.5 (12,2÷49). **Table 1** describes the demographic and clinical features of the two groups.The two groups are not comparable because sex and age were significantly different: our medical staff was 86% females, while patients were 63% females; the employees in our department were much younger than our patients: 45 years median age compared to 59 years in the patients group.

**Table 1:** Demographic and biological features of the patient group and the medical staff group

Parameter	Patients (325)	Medical staff (58)	p-value patients vs. medical staff
Gender F (%)	63%	86%	P<0.001
Age*	59 (17÷88)	45(25÷60)	P<0.001
BMI*	25.5 (12.2÷49)	25.7(16.6÷28.7)	P=0.874
Urban area (proportion)	79%	86%	P=0.283
Hemoglobin*	13.5 (6.7÷ 16.6)	12.2 (10.4÷14)	P<0.001
Serum ferritin (g/L)*	124.95 (4.1÷10963)	37.05 (4.3÷306.6)	P<0.001
Transferrin saturation (%)*	22.9 (4÷60.6)	24 (1.7÷94.3)	P=0.169
Serum iron (mcg/dL) *	85 (15.8÷216)	82 (8÷321)	P=0.950
Serum transferrin (g/L)*	2.7 (1.8÷4)	2.5 (0.9÷4.3)	P=0.001

* median with range

**Fig. 2:**
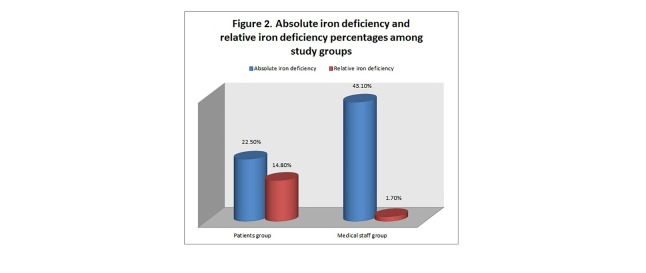
Absolute iron deficiency and relative iron deficiency percentages among study groups

The prevalence of absolute iron deficiency was 22.5% in patients and 43.1% in medical staff (p=0.002), while the relative iron deficiency was present in 15% of patients and 1.7% of medical staff (p=0.002)(**Figure 2**).

Iron deficiency anemia was encountered only in 20 cases out of 325 patients (6.2%).

Gastrointestinal bleeding at admission was found in 8% (24/325) of the patients, of whom 83% (20/24) were anemic.

Regarding concomitant medications, 7% of our patients took acenocoumarol, 14% aspirin and 30% admitted the consumption of NSAIDs. Among patients, the absolute iron deficiency was significantly associated with the female sex (p=0.002) and pre-menopausal status (p=0.01), but it was not associated with the urban/rural area. We found no association with age (p=0.19), BMI (p=0.1), NSAID (p=0.73), aspirin (p=0.77) or acenocoumarol (p=0.84) consumption (**Table 2**).

**Table 2:** Correlation between the demographical parameters and the absolute iron deficiency

Variable	Absolute iron deficiency Y	p-value
Sex F*	27.5%	0.002
Sex M*	7.7%	
Pre-menopausal status*	21%	0.014
Post-menopausal status*	46.4%	
Urban area*	13.9%	0.295
Rural area*	21.7%	

* number (percent), compared by Fisher’s exact test

**Table 3** illustrates the prevalence of absolute iron deficiency according to the diagnosis. This situation was more frequently present in patients with inflammatory bowel diseases (62.5%) and colorectal cancer (52%), whereas it was rarer in other types of cancers (0÷50%). In functional digestive diseases, the absolute iron deficiency had a prevalence of 25-29%, and it was very rarely encountered in patients with liver cirrhosis and other chronic liver diseases, as in peptic diseases (chronic gastritis, peptic ulcer disease and gastro-oesophageal reflux disease). Although there were big differences, they did not reach a statistical significance, probably because of the lack of statistical power.

**Table 3:** Prevalence of absolute iron deficiency, of relative iron deficiency and iron deficiency anemia according to diagnosis in our patients

Diagnosis- number of patients	Prevalence of absolute iron deficiency (proportion and number)	Prevalence of relative iron deficiency (proportion)	Prevalence of iron deficiency anemia (proportion)
Functional dyspepsia- 7	29%- 2	0%	0%
Alcoholic liver cirrhosis- 27	0.4%-1	37%-10	0.4%-1
Chronic hepatitis regardless of etiology- 43	21%-9	7%-3	0%
Peptic diseases- 40	15%-6	7.5%-3	10%-4
Liver steatosis- 32	25%- 8	0%	0%
Diverticulosis- 5	40%- 2	0%	20%-1
Inflammatory bowel diseases-16	62.5%-10	0%	19%- 3
Viral liver cirrhosis- 56	9%- 5	9%-5	5.4%-3
Post-operative surgical adhesions- 6	17%- 1	50%-3	0%
Irritable bowel syndrome- 28	25%- 7	7%-2	0%
Colorectal cancer- 21	52%- 11	38%-8	19%-4
Hepatocellular carcinoma- 7	0%-0	57%- 4	0%
Pancreatic cancer- 6	33%-2	67%- 4	33%-2
Gastric cancer- 2	50%-1	0%	0%
Other cancers- 7	43%- 3	43%-3	28.5%-2
Benign gastrointestinal tract polyps- 10	40%- 4	20%-2	0%
Haemorrhoids- 5	20%-1	0%	0%
Chronic pancreatitis- 2	0%-0	0%	0%
p-value	0.426	0.014	0.01

The relative iron deficiency was significantly associated with the diagnosis of alcoholic liver cirrhosis (37% prevalence) and cancer, especially hepatocellular carcinoma (57%) and pancreatic cancer (67%) (p=0.01) (**Table 3**). Unexpectedly, we also found this specific iron deficiency in a few patients with peptic diseases, chronic hepatitis, post-operative surgical adhesions, irritable bowel syndrome, but the explanation in these cases were the co-morbidities: chronic pyelonephritis, rheumatoid arthritis, recent abdominal surgery, ankylosing spondylitis (**Table 3**). Also, the advanced age was a strong predictor: the subjects with relative iron deficiency had a median age of 70 years (min. 58, max.88), while those without relative iron deficiency had a median age of 59 years (min. 17, max.84), p=0.001 (Mann-Whitney U test).

Iron deficiency anemia was associated with advanced age (p=0.03) and diagnosis of cancer, erosive gastritis and inflammatory bowel disease (p=0.01) (**Table 3**).

## Discussion

Iron deficiency anemia was a relatively frequent reason for admission in our department (6.2%), but studies regarding the prevalence and significance of iron deficiency in gastrointestinal and liver diseases are quite a few.

That is why the aim of this study was to explore the "state of iron" in patients admitted to our department for 2 weeks.

We found a 22.5% proportion of absolute iron deficiency in our patients, which was much lower than the rate reported by Mearin et al. (54%), but these authors used a different definition of iron deficiency (ferritin < 30ng/ml or transferrin saturation < 16%), and the patients analyzed by these authors were admitted with more severe gastrointestinal diseases than our subjects [**22**]. Very recent data reported by Fonseca et al. indicated a prevalence of absolute iron deficiency of 58% and iron deficiency anemia of 41% of patients admitted to an Internal Medicine ward, but these patients were also diagnosed with more severe diseases than our patients: ≥65years old (80%), with hypertension (63%), moderate chronic kidney disease (CKD) (43%), and heart failure (41%) [**23**].

We included in our studies patients with continuous hospitalization and one-day hospitalization, and the spectrum of diseases among them was quite diverse, as **Table 1** illustrates: 25% were admitted with liver cirrhosis, 13% with chronic hepatitis, 12% because of peptic diseases, 10% with liver steatosis; 11% had irritable bowel syndrome and functional dyspepsia. Digestive cancers represented an important proportion: 13% of our patients, most of them being patients with colorectal cancer (half of them). Less often, patients were admitted for inflammatory bowel diseases, diverticulosis, post-operatory adhesions, benign polyps, chronic pancreatitis or hemorrhoids.

In an adult population in Catalonia, Spain, the prevalence of iron deficiency was 5.6%, and was especially frequent in women aged 50 years or younger (14.8%) [**24**]. In a study assessing the iron status in an adult population in Finland, iron deficiency was found in an increased proportion in younger women (20%) compared to older women (11%) [**25**].

Surprisingly, in our medical staff (physicians, nurses), the absolute iron deficiency was an even bigger problem than it was in our patients, reaching 43.1%, and that could be explained by the fact that most of them were women (86%), more than 75% were pre-menopausal, but all of them were unaware of this iron depletion, that it might have had significant long-term consequences. Heavy menstrual bleeding (HMB), which is a gynecologic problem that affects up to 30 % of premenopausal women [**26**][**27**], is the most frequent cause of iron deficiency in our medical staff.

As it would have been expected, the absolute iron deficiency was associated with the female sex and pre-menopausal setting, but we found no significant correlation with age, BMI and with the consumption of aspirin, NSAIDs or acenocoumarol.

Among patients with inflammatory bowel diseases (IBD), the iron depletion was very frequently encountered (62.5%), as it was in people with colorectal cancer (52%) or other types of cancers, very different from other gastrointestinal diseases, but the study was underpowered, because of the relatively low number of cases. Many studies from the literature investigate the issue of iron deficiency anemia in inflammatory bowel diseases, and, in a very recent one, the proportion of absolute iron deficiency was quite similar to our finding (53%) [**30**].

Identifying ID in patients with IBD seems important because data from the literature are showing that intravenous iron treatment may improve QoL in nonanemic, but iron deficient IBD patients [**32**][**33**].

We found a 19% prevalence of iron deficiency anemia among our patients with IBD, which was lower compared to other studies (21-40%) [**28**][**31**][**33**][**34**]. That difference might be explained by the fact that our study included a much smaller number of patients.

Iron metabolism disorders in patients with liver cirrhosis are quite frequent. Other researchers found iron deficiency in 28.6% of cirrhotics and iron overload in 18.9% of them [**36**]. Our data showed a rare prevalence of absolute ID in viral liver cirrhosis (9%), while in alcoholic cirrhosis the iron deficiency was very rare (0.4%), but these differences might be explained by the low sample size. Iron deficiency in patients with liver cirrhosis is mainly due to acute or chronic GI hemorrhage, having multiple causes (portal hypertension, impaired blood coagulation) [**42**]. According to our findings, the relative ID is much more prevalent in alcoholic liver cirrhosis (37%). In this situation, because of chronic inflammation, the absorption of iron from enterocytes is blocked due to the elevated serum hepcidin level. It would have been interesting to explore the prevalence of iron overload in our cohort of patients, but, unfortunately, we did not have enough data. The iron overload is defined as serum ferritin higher than 300 ng/ml in males and postmenopausal females, and higher than 200 ng/ml in premenopausal females [**36**]. But serum ferritin is also increased in acute and chronic inflammation, and, in order to diagnose the iron overload, we would also need a measurment of the C-reactive protein level and total iron binding capacity in the serum. Our data showed increased levels of ferritin in 22% of subjects, that is 72 out of 325: in 17 cases we identified acute or chronic inflammation: urinary tract infection, rheumatoid arthritis, acute cholecystitis. The proportion of patients with increased serum level of ferritin is 71% in alcoholic liver cirrhosis, 23% in viral liver cirrhosis, 22% in chronic hepatitis, 12% in liver steatosis, but in these cases, it is impossible to differentiate between iron overload and inflammation. We have also encountered very high levels of ferritin in pancreatic cancer (67%) and other cancers (liver metastases from ovarian cancer, uterine cancer or liver metastases of unknown origin), and in these cases the explanation is inflammation.

Our data show a 52% prevalence of absolute ID and 19% for ID anemia in patients with colorectal cancers, which is a lower prevalence than literature data (50-60%), but the explanation for this difference comes from the fact that half of our patients are already treated (operated) [**37**][**38**][**39**][**40**]. The cause of iron deficiency is mainly occult or overt lower GI bleeding, but the proportion of relative ID is also high in our patients (38%), which suggests that the inflammation is also a frequent cause of anemia in these patients. Ferritin is an important serum marker in the setting of suspicion of colorectal cancers, as some researchers have found that its level above 100 mcg/L could rule out colon cancer, but not gastric or rectal cancer in patients with involuntary weight loss [**44**].

Diverticulosis is a rare pathology among our patients, but the frequency of absolute ID and ID anemia is quite high: 40% and 20% respectively, similar to other researchers’ findings [**41**][**42**].

One important question for the gastroenterologist is if the patients with iron deficiency without anemia would benefit from an endoscopic examination, and the results of a recent study showed that the prevalence of gastrointestinal lesions in patients with and without anemia was similar but malignancy was eight times less frequent in the iron deficiency group [**30**]. Therefore, systematic endoscopic evaluation in patients with iron deficiency is questionable [**30**].

To treat ID, most of the guidelines have preferentially recommended the oral route, if possible, particularly in women in the pre- or postpregnancy period. Iron supplementation should be administered intravenously in patients with chronic kidney disease (CKD) and chemotherapy-induced anemia. Treatment targets for ID included an increase in hemoglobin concentrations to 10-12 g/dL or normalization and serum ferritin >100 μg/L [**31**].

Our study is addressing the issue of iron deficiency in gastroenterology and hepatology, for which there are limited data in the literature, but it has some limitations. The major limitation is the relatively low number of subjects (325), leading to a reduced number of patients with each disease. Another limitation is that we tried to define a control group from the medical staff, but, unfortunately, these groups were not statistically comparable, mainly because of sex and age. Finally, because we introduced in the study patients with one-day hospitalization, our results are difficult to compare with other literature studies that have enrolled patients with more severe diseases.

In conclusion, iron deficiency was an important issue in our patients, reaching 22.5% prevalence of absolute ID and 15% relative ID. The absolute ID was even more important in the medical staff, the prevalence rate reaching 40%. We should always think about the "state of iron" in our patients, as even without anemia, ID can have a substantial impact on physical and cognitive function and quality of life (e.g., fatigue). There is a need for regular assessment of iron status.

**Acknowledgment**

This study is a result of a grant given by Vifor Pharma Romania.

Blood samples were analyzed in an external laboratory (Synevo) with the financial support of Vifor Pharma Romania.

Vifor Pharma Romania had no other involvement or control over the conduct of this research, excepting the above-mentioned support in determining the biochemical parameters.

**Disclosures**

The authors declare that there is no conflict of interest.

**Financial support statement:**

This work was supported by a Vifor Pharma Grant received in 2016.
